# Randomized controlled trial on the comparison of chest tube drainage and needle aspiration in the treatment of primary spontaneous pneumothorax

**DOI:** 10.12669/pjms.346.16126

**Published:** 2018

**Authors:** Ali Ramouz, Mohammad Hossein Lashkari, Sanam Fakour, Seyed Ziaeddin Rasihashemi

**Affiliations:** 1 *Ali Ramouz, Research Fellow, Dept. of General Surgery, AJA University of Medical Sciences, Tehran, Iran*; 2 *Prof. Mohammad Hossein Lashkari, Dept. of General Surgery, AJA University of Medical Sciences, Tehran, Iran*; 3 *Sanam Fakour, Research Fellow,Dept. of General Surgery, Tabriz University of Medical Sciences, Tabriz, Iran*; 4 *Seyed Ziaeddin Rasihashemi, Assistant Professor, Dept. of Cardiothoracic Surgery, Tabriz University of Medical Sciences, Tabriz, Iran*

**Keywords:** Chest tubes, Pain, Pneumothorax, Recurrence

## Abstract

**Objectives:**

To evaluate the efficacy of the chest tube drainage (CTD) and the needle aspiration (NA) in the treatment of primary Spontaneous pneumothorax (SP).

**Methods:**

In a randomized controlled trial, seventy patients suffering SP were divided equally into two subgroups, as follows: (A) CTD and (B) NA. The immediate and one-week rate of the treatments was the primary endpoints. Postoperative complications, length of hospital stay and incidence of pneumothorax recurrence during one-year follow up were also recorded.

**Results:**

The immediate success of treatment was 68.5% and 54.2% of patients in CTD and NA groups, respectively that showed no significant difference between study groups (P: 0.16). The complete lung expansion after one week observed in 32 (91.4%) of NA group and 33 (94.2%) patients in CTD group (P: 0.5). Pneumothorax recurrence was detected in 13 patients (4 in NA and 9 in CTD group) (P: 0.11). Mean pain intensity was significantly lower in the NA group at the first hour after the procedure, the first postoperative day and the first week after the intervention (P< 0.001).

**Conclusion:**

Needle aspiration (NA) can be applied as a first step treatment in patients with primary SP, considering its advantages.

## INTRODUCTION

Spontaneous pneumothorax (SP) is a common complication, which refers to the presence of air in the pleural space which is not followed by an injury such as a rib fracture.^1^ The most regularly used treatments for primary SP are conservative treatments including Chest tube drainage (CTD) and needle aspiration (NA).^2^ CTD is accepted as the standard treatment for SP considering its high efficacy that can expand the lungs of the patients and relieve the symptoms.^3^ However, aspiration via a needle or NA has been welcomed by physicians because of its easy application and quick benefits, as well as reducing patient irritation.^2^,^4^

Not many studies have compared the current therapeutic approaches and their effectiveness, which have contradictory results.^5^-^7^ We compared the two standard methods of primary SP treatment, including CTD and needle aspiration, to evaluate the efficacy of the treatments considering advantages and disadvantages such as pneumothorax relapse.

## METHODS

### Patients

A single-blinded, two-central randomized controlled trial carried out between September 2017 and June 2018, in thoracic and general surgery wards, Tabriz Imam Reza and Tehran Imam Reza hospitals, Tabriz University of Medical Sciences and AJA University of Medical Sciences. The study protocol was approved by ethics committee of the AJA University of Medical Sciences (Committee reference number: IR.AJAUMS.REC.1396.24), and registered in ClinicalTrials.gov (Trial registration: NCT03293199, Verified: September 2017). All patients older than 18 years old with symptomatic primary SP (SP), enrolled. Light pneumothorax size calculation formula was, as follows: [1-((collapsed lung diameter)^3^/(involved hemithorax diameter)^3^)].^8^

Patients suffering tension pneumothorax, bilateral severe respiratory failure, and bilateral pneumothorax or indicated for mechanical ventilation excluded. Written informed consent obtained from all of the patients.

With due attention to the success rate of 18% provided in a previous study,^9^ comparing primary SP treatment procedures and the study power of 80% and the confidence coefficient of 0.05, study population calculated to include 64 patients, which increased to 70 patients with taking 10% of falling risk into consideration. Randomization performed using Randlist software.

### Methods

Demographic data including patients’ age, gender, body weight, body height and body mass index (BMI) and history of cigarette smoking (reported as the packed year in smoking individuals), recorded, as well as time interval between symptoms onset and hospital admission. Before the intervention, patients underwent chest radiography to confirm the primary SP diagnosis. During hospital admission, as a standard treatment, 3 L/min-1 supplementary oxygen administered for all patients. Furthermore, patients received pneumothorax treatment via one of the methods ((A) CTD and (B) NA) based on the study group. Repeated CXR performed at postoperative day (POD) one and seven, to evaluate complete lung expansion and confirm successful treatment. Postoperative pain intensity evaluated after the intervention, at discharge and one-week later a using Visual Analogue Scale (VAS) of 0-10, that 0 showed no pain and 10 indicated the worst pain.

The immediate and one-week success rate of the treatments was defined as the primary endpoint, as well as the one-year recurrence rate. Operation time was calculated between the time of skin incision or needle insertion and completion of the air aspiration with a maximum volume of 3.5 liters. Postoperative complications including bleeding or pleural effusion, interval till pneumothorax recurrence and length of hospital stay were also recorded.

### Surgical Procedure

In CTD group (Group-A), while the patient was positioned in the supine position and subsequent to local anesthesia, F16 or F20 (based on patients’ physical status) sterile plastic tube implemented at the level 4th or 5th intercostal space through the midaxillary line and the tube connected to two-chest water seal canisters. Cease of bubbling in water seal canisters with symptoms resolution or pneumothorax size <10% were considered an adequate response to CTD treatment. CT remained for 24 hours, and patients discharged after CXR repeated 24 hours following to CT removal.

However, in NA group (Group-B), patients positioned semi-supine and local anesthesia administered. Subsequently, G16 intravenous angiocath inserted through the midclavicular line at the level 2nd or third intercostal space and after penetration of parietal pleura by catheter. The air suction performed using a 50-ml syringe till the end of the air aspiration or up to 3.5 liters of air suctioning. Without catheter removal, patients underwent CXR and discharged six-hour later in case of symptoms resolution, and lung expansion with a narrow rim of pneumothorax (<20%). However, in the case of poor lung expansion, re-aspiration performed via the primary catheter. If more than 4 liters of air was aspirated or lung expansion was failed after the second try for needle aspiration, patients underwent CTD implementation.

Finally, patients who did not have successful treatment either by NA nor CTD, patients underwent chemical pleurodesis with talc powder injection via chest tube or video-assisted thoracoscopic surgery (VATS) at the physicians’ discretion.

### Statistical analysis

The student T test used for comparing quantitative variables between groups and the Chi-square Test for comparing two qualitative variables in each time, and between different times. The level of significance was set at 0.05, and all results were expressed by frequency (percent) for qualitative variables and Mean±SE for quantitative variables. The two-way repeated measures ANOVA is used to determine if there was a statistically significant interaction effect between two treatment approaches including CTD and NA on patients’ pain intensity during one-week follow up period.

## RESULTS

Patients demographic data are listed in Table-I. According to pneumothorax treatment approach, Considering patients’ age, gender and cigarette smoking history and smoking amount, no significant differences between CTD and NA groups (Table-I).

**Table-I:** Patients demographic data and characteristics.

Variable		Technique		Pv
		CTD group (n:35)	NA group (n:35)
Age		49.83±7.48	48.87±9.37	0.63
Gender	Female	6(8.6%)	4(5.7%)	0.36
	Male	29(41.4%)	31(44.3%)
BMI (Kg/m2)		22.30±2.63	21.20±2.38	0.07
Current smoker		11(15.7%)	16(22.9%)	0.16
Mean smoking amount (pack year)		5.17±0.84	4.84±1.02	0.38
Pneumothorax size		49.28±9.64	53.82±12.51	0.09
Involved hemithorax	Left	17(24.3%)	13(18.6%)	0.23
	Right	18(25.7%)	22(31.4%)
Symptoms onset to treatment (hours)		17.73±4.69	19.57±3.39	0.06

**Abbreviations:** CTD: CTD, NA: Needle Aspiration, BMI: Body Mass Index, Pv: P-value.

Procedure duration, successful treatment rates, duration of hospital stay, pneumothorax recurrence and post-interventional complications are summarized in Table-II. Mean operative times were significantly lower in the CTD group compared to the NA group (P< 0.001). The immediate success of treatment observed in 24 (68.5%) and 19 (54.2%) patients in CTD and NA groups (P: 0.16). Concerning, the one-week success rate of each treatment, complete lung expansion observed in 32 (91.4%) of NA group, as well as 33 (94.2%) patients in CTD group (P: 0.5). While, of 32 patients who had successful treatment via needle aspiration, pneumothorax resolved in 19 (59.4%) patients during the first attempt, however, in 13 (40.6%) patients the second attempt was needed to obtain complete lung expansion. Treatment flow of the patients in the NA group has been shown in Fig.2. Overall, five (7.1%) patients showed no improvement in pneumothorax symptoms and lung expansion after NA and CTD procedures (2 in CTD group and 3 in the NA group). Hence, four patients received chemical pleurodesis using talc powder (3 NA and 1 CTD), and one patient underwent pleurectomy via VATS.

**Table-II:** Procedures characteristics, success rate, hospital admission and postoperative complication.

		Technique		Pv
		CTD group (n:35)	NA group (n:35)
Operation time (min)		21.63±4.32	34.87±9.47	<0.001
Success rate	Immediate	24 (68.5%)	19 (54.2%)	0.16
	One-week	33 (94.2%)	32 (91.4%)	0.5
Hospital admission (day)		4.15±1.07	1.39±0.34	<0.001
One-year recurrence		4/32 (12.5%)	9/33 (27.2%)	0.11
Time to recurrence (weeks)		18.57±7.46	16.23±8.52	0.64
Short-term Complications	Overall	6 (17.1%)	1 (2.8%)	0.053
	Wound infection	1 (2.8%)	0	0.5
	Bleeding	3 (8.5%)	1 (2.8%)	0.33
	Subcutaneous emphysema	2 (5.7%)	0	0.24

**Abbreviations:** CTD: CTD, NA: Needle Aspiration, Pv: P-value.

**Fig.1:**
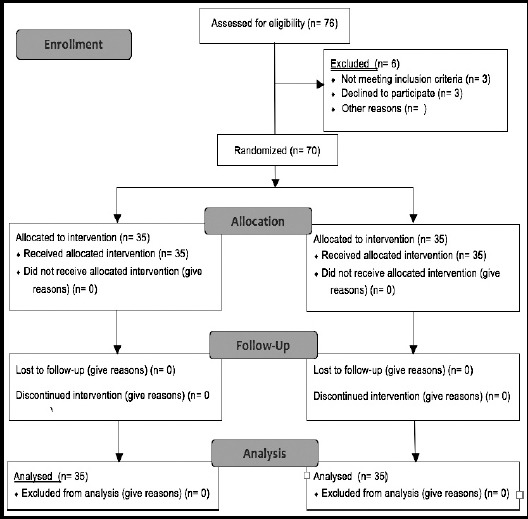
CONSORT 2010 study flow diagram.

**Fig.2:**
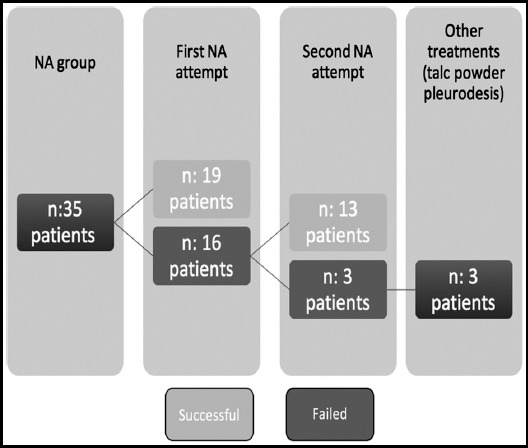
Visual Analogue Scale (VAS) pain scores during one week follow up period.

### Abbreviations: NA: Needle Aspiration.

Complications after each intervention are listed in Table-II. Although our results showed that overall rate of post-procedure complications was higher in patients undergone CTD, the prevalence of overall complications was slightly not significant (P:0.05). However, mean duration of hospital stay was significantly lower in NA group compared to CTD group (P< 0.001).

Pneumothorax recurrence was detected in 13 patients (18.5%), which showed no significant difference between CTD and NA groups regarding its incidence (P:0.11) (Table-II). Comparing the mean recurrence interval revealed no significant difference between study groups (P:0.64). Of thirteen patients with recurrent pneumothorax, nine patients received chemical pleurodesis via talc powder and 4 patients underwent VATS pleurectomy due to the physician discretion.

**Fig.3:**
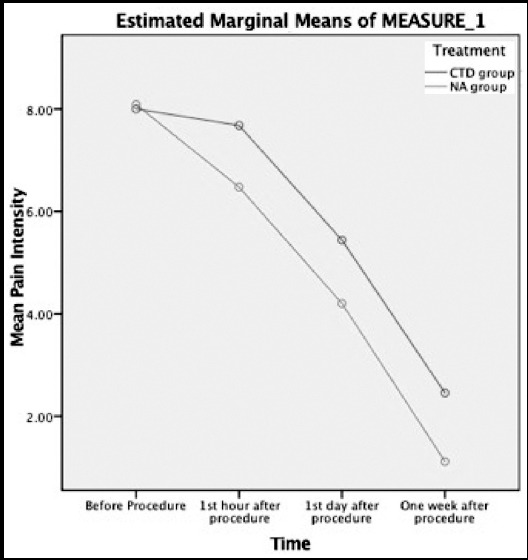
Repeated measure test to evaluate pain intensity change during follow-up time points.

Using a two-way repeated measures ANOVA, there was a statistically significant interaction between treatment and time on pain intensity, F(2.52, 146.35) = 15.95, p< 0.001 (Fig.3). Mean pain intensity was not statistically significantly different in the CTD group compared to the NA group at the pre-interventional state (p=0.489). However, mean pain intensity was statistically significantly different in the CTD group compared to the NA group at the first hour after the procedure (p< 0.001). Mean pain intensity was also statistically significantly different in CTD group compared to that of NA group at the first POD (p< 0.001), as well as, the first week after procedure (p< 0.001).

**Table-III:** Pre- and Post-operative total and ionized calcium in HS and LSJ groups.

Variable		Total						Pv

		Technique	CTD group (n:35)	NA group (n:35)	Mean Difference	95% CI	Independent t-test	Repeated Measure

		Time	Mean±S.D.					
Pain intensity (VAS)	Total (mg/dL)	Pre-intervention	8±0.89	8.08±1	-	-	0.48	<0.0001
		After Intervention	7.68±0.97	6.47±1.04	1.203	0.87-1.53	<0.001	
		First POD	5.44±1.34	4.2±1.44	1.237	0.82-1.65	<0.001	
		Seventh POD	2.46±0.82	1.12±0.97	1.33	1-1.66	<0.001	

***Abbreviations:* CTD:** CTD, **NA:** Needle Aspiration; **POD:** Postoperative day; **Pv:** P-value. **SD:** Standard Deviation; **CI:** Confidence interval.

## DISCUSSION

To our knowledge, this is for the very first time that a multi-center study compares two approaches in the treatment of the primary spontaneous on the homogeneous population in our region. According to the literature, in patients with the first episode of the SP treatment goal mainly focuses on removal of the accumulated air in pleural space; however, recurrence prevention is the primary concern in patients with secondary or relapsing pneumothoraxes.^3^,^10^

In the current randomized controlled trial, results showed no significant difference between NA and CTD techniques concerning immediate and one-week success rate of the treatment; however, patients who benefited NA technique had a shorter hospital stay accompanied with lower pain intensity. In an early study by Andrivet et al. evaluating 61 patients with SP, although results showed no significant difference in hospital admission period and rate of pneumothorax recurrence, CTD technique resulted in better success rate and outcomes.^11^ The prolonged hospital stays in NA patients evaluated in Andrivet study regard to 72 hours delay of the intervention in some patients, which led to a bias in the mean duration of hospital admission.^11^ Also, they applied more tolerated criteria to evaluate CTD failure such as consideration of the 10-day treatment period to define CTD failure. Thus, we believe immediate NA not only provides higher or similar success rate compared to CTD in primary SP treatment but also results in significant reduction of the patients’ hospitalization. Similarly, in a pilot study conducted by Noppen et al., authors suggested equal immediate and one-week success rate for NA and CTD procedures in the treatment of the primary SP, as well as one-year recurrence rate for pneumothorax.^12^ The authors decided to discharge patients after the NA; besides, we believe rapid discharge without hospitalization is almost inevitable in patients who undergo CTD implementation; thus it is evident that CTD patients should have significantly higher hospital admission rate compared to NA group. In a similar study, Ayed et al. provided parallel outcomes to Noppen et al. study, as they reported a higher hospitalization rate and more extended hospital stay for patients who underwent CTD compared to NA patients.^13^ However, in our study, we did not consider immediate discharge, and NA group patients admitted for at least 24 hours to evaluate complete lung expansion. Our results demonstrated that NA procedure in primary SP patients decreases the duration of hospital stay compared to CTD implementation, which was in coincidence with Ayed et al.^13^ results. On this basis, it seems that the necessity and benefits of the hospital admission in primary SP patients who receive NA procedure should be evaluated in further studies; however, our results showed that NA procedure results in shorter hospital stay compared to CTD. A systematic review has compared the simple aspiration against intercostal tube drainage in primary SP in adults, reported no significant difference between two techniques about immediate success rate, early failure rate, duration of hospital stays and one-year success rate, but stated that NA is accompanied with a reduction in the rate of hospital admission.^14^ However, in this review, only a single trial has met the inclusion criteria to be included in the review process, and no meta-analysis has been conducted to provide more accurate results and conclusion especially regarding the duration of hospital stay. Thus, it does not obtain extendable results to prevent more studies to be designed.

Concerning post-interventional complications, seven (10%) patients developed complications in our study, whereas only one patient belonged to the NA group who suffered bleeding. Several studies have reported no complication for NA procedure while CTD technique can lead to mild and severe complications such as pleural effusion, subcutaneous emphysema, empyema, and pneumonia. We suppose inappropriate needle insertion may have led to bleeding in the only patients with complications in NA group. However, none of the patients had hospital admission or life-threatening events following to the acquired complications. Besides, the only disadvantage of NA technique in the present study while comparing to CTD, was more prolonged duration of the procedure.

Although Ayed et al. recorded analgesics consumption between primary SP patients underwent NA and CTD treatment, to compare pain severity between study groups and showed fewer analgesics consumption in NA patients,^13^ the present study was first to determine the pain severity in treated primary SP patients using VAS. We obtained pain intensity objectively in four time-points as follows, admission time, one hour after the procedure, first POD and seventh POD. No significant difference observed in patients’ pain intensity at the trial beginning, but postoperatively, pain intensity reduction was significantly higher in patients who underwent NA procedure compared to CTD group, which remained constant till the end of the one-week follow-up.

### Limitations of the study

First, different surgeons performed the procedures which may have caused bias in the evaluation of the operative time described in the present study, however, the surgeons had approximately similar clinical experience. Second, the patients cannot be blinded to the allocated study group.

Our results demonstrated that although NA and CTD have similar short-term and long-term efficacy and safety in the treatment of the patients with symptomatic primary SP, NA application decreases hospital stay duration and induces a rapid reduction in pain intensity of the patients compared to CTD technique. Thus, we suggest NA as a first step treatment in patients with primary SP, considering its advantages.

### Author’s Contribution

**AR** conceived, designed and did the statistical analysis and edited the manuscript.

**MHL, SF and SZR** collected the data, interpreted the analysis and writes the manuscript.

**AR and MHL** did the final revision and approval of the manuscript.
